# Histopathological Effects of Titanium Dioxide Nanoparticles and The Possible Protective Role of N-Acetylcysteine on The Testes of Male Albino Rats 

**DOI:** 10.22074/ijfs.2018.5389

**Published:** 2018-06-20

**Authors:** Amir M. Bassam Elnagar, Abdelnasser Ibrahim, Amro Mohamed Soliman

**Affiliations:** 1 Department of Histology, Faculty of Medicine, Al-Azhar University, Assiut, Egypt; 2 Department of Pathology, Insaniah University, Kuala Ketil Kedah, Darul Aman, Malaysia; 3 Forensic Unit, Department of Pathology, National University of Malaysia Medical Centre, Jalan Yaakob Latif, Bandar Tun Razak, Kuala Lumpur, Malaysia; 4 Department of Forensic Medicine and Clinical Toxicology, Faculty of Medicine, Al-Azhar University, Cairo, Egypt; 5 Department of Anatomy, National University of Malaysia Medical Centre, Jalan Yaakob Latif, Bandar Tun Razak, Kuala Lumpur, Malaysia

**Keywords:** N-acetylcysteine, Oxidative Stress, Testis, Titanium Dioxide, Toxicity

## Abstract

**Background:**

Titanium dioxide (TiO_2_) is a white pigment which is used in paints, plastics, etc. It is reported that TiO_2_ induces oxidative stress and DNA damage. N-acetylcysteine (NAC) has been used to fight oxidative stress-induced damage in different tissues. The objective of this study was to evaluate the toxic effects of orally administered TiO_2_ nanoparticles and the possible protective effect of NAC on the testes of adult male albino rats.

**Materials and Methods:**

In this experimental study, 50 adult male albino rats were classified into five groups. Group I was the negative control, group II was treated with gum acacia solution , group III was treated with NAC, group IV was treated with TiO_2_nanoparticles, and group V was treated with 100 mg/kg of NAC and 1200 mg/kg TiO_2_nanoparticles. Total testosterone, glutathione (GSH), and serum malondialdehyde (MDA) levels were estimated. The testes were subjected to histopathological, electron microscopic examinations, and immunohistochemical detection for tumor necrosis factor (TNF)-α. Cells from the left testis were examined to detect the degree of DNA impairment by using the comet assay.

**Results:**

TiO_2_nanoparticles induced histopathological and ultrastructure changes in the testes as well as positive TNF-α immunoreaction in the testicular tissue. Moreover, there was an increase in serum MDA while a decrease in testosterone and GSH levels in TiO_2_nanoparticles-treated group. TiO_2_resulted in DNA damage. Administration of NAC to TiO_2_- treated rats led to improvement of the previous parameters with modest protective effects against DNA damage.

**Conclusion:**

TiO_2_-induced damage to the testes was mediated by oxidative stress. Notably, administration of NAC protected against TiO_2_’s damaging effects.

## Introduction

Titanium dioxide (TiO_2_), also recognized as titanium oxide or titania, is a naturally occurring oxide of titanium which is commonly used as a colouring pigments to provide a white colour in products such as dyes, plastic, paper, and foods (1). TiO_2_ either alone or in mixtures, is broadly used for a wide range of medical procedures. However, in case of imbalanced biological conditions such as low pH, TiO_2_ devices can release enormous amounts of particles at both micrometer and nanometer levels (2).

The lethal effects of nanoparticles can be accredited to their small size and hence outsized surface area which results in increased rates of chemical reaction and infiltration into the cells interfering with numerous subcellular physiological mechanisms (3). For instance, studies presented that nanoparticles may infiltrate into nuclei and later may interfere with the organization and functions of DNA (4). Furthermore, TiO_2_ nanoparticles may produce reactive oxygen species (ROS) resulting in cell toxicity (5). Previous studies reported that TiO_2_ nanoparticles elicited different antagonistic cellular properties including DNA injury (6).

Previous studies have established that TiO_2_. cles may pass into the cells of the reproductive system and induce damage (7). Takeda et al. (8) declared that TiO_2_ nanoparticles were found in the testes and brain, which indicated that TiO_2_ nanoparticles may penetrate both blood-testis and blood-brain barriers. Komatsu et al. (9) confirmed that TiO_2_ nanoparticles were absorbed in Leydig’s cells leading to disruption of cellular proliferation and dysregulation of the expression of heme oxygenase- 1 (HO-1), a steroidogenic regulatory protein, which regulates mitochondrial cholesterol transfer. This implies that long-period exposure to small doses of TiO_2_ nanoparticles should not be ignored and the possible risks that these particles may impose to reproductive health should be considered, particularly in those who are occupationally exposed to TiO_2_ nanoparticles.

N-acetylcysteine (NAC) is an antioxidant and free-radical scrounger. It acts as a cysteine contributor and upholds or even upsurges the intracellular levels of glutathione (GSH) (a tripeptide which guards cells against toxins). NAC is characterized by its antioxidant ability through releasing sulfhydryl groups which in turn, reduce ROS levels and possess the ability to reduce oxidative stress directly, suppress the nuclear factor kappa b (NF-.B) inflammation pathway and inflammatory cytokines secretion, and enhance the GSH production (10). NAC was proven to fight oxidative stress-induced injury in several tissues. For instance, administration of NAC suppressed oxidative stress-induced cellular damage in different injury models including head injury, endotoxin-induced lung damage, liver injury, and heart disorders (11). In order to explore the reproductive toxicity of TiO_2_ nanoparticles, the present study was conducted. The toxic effects of orally administered TiO_2_ nanoparticles were evaluated in the testes of adult albino rats through histopathological, ultrastructural examinations, immunohistochemical detection of tumor necrosis factor (TNF)-a, measurement of total testosterone and oxidative stress levels, and comet assay. Moreover, the possible protective effects of co-administration of NAC and TiO_2_ nanoparticles were assessed.

## Materials and Methods

The current experimental study was carried out in Animal Laboratory, Zagazig University. TiO_2_ nanoparticles (Titanos, China) were nanopowder of 21 nm size with =99.5% purity dissolved in gum acacia solution. NAC was purchased from SEDICO, Egypt. Fifty male albino rats weighing 150-200 g were obtained from the Animal House, Faculty of Medicine, Zagazig University. The study was conducted in accordance with the guidelines of the Ethics Committee for Research of Zagazig University. The rats were divided into 5 groups. Group I was the control group that received no treatment. Group II was treated with 1 ml of 5% gum acacia solution (the solvent used for titanium dioxide) by oral gavage once daily for 12 weeks. Group III was orally treated with 100 mg/kg of NAC once daily for 12 weeks. Group IV was orally treated with 1200 mg/ kg of TiO_2_ nanoparticles once daily for 12 weeks. Group V was orally treated with a combination of 100 mg/kg of NAC and 1200 mg/kg of TiO_2_ nanoparticles once daily for 12 weeks.

For histopathological analysis, the left testis was fixed in Bouin’s solution and the tissue was processed and embedded in paraffin blocks for preparation of 5-µm thick sections. Sections were stained with Haematoxylin and Eosin and examined by light microscopy. Ultrastructural examination of the left testis was conducted using the Transmission Electron Microscope (TEM). The analysis was performed according to the method described by Glauret and Lewis (12). The stained sections were examined by TEM in Electron Microscope Center in the department of histology, Faculty of Medicine, Zagazig University. Immunohistochemistry of the left testis was performed using labeled streptavidin-biotin (LSAB) technique. The deparaffinized sections were incubated with hydrogen peroxide to block the endogenous peroxidase. Then, sections were incubated with primary antibodies for TNF-a (rabbit polyclonal TNF-a antibody). Then, sections were incubated with the secondary antibodies and peroxidase-labeled streptavidin. Staining was completed by incubation with substrate chromogen, which resulted in the brown-colored precipitates at the antigen sites. 

Blood was collected from the tail vein then it was centrifuged to collect serum. Total testosterone level was measured by enzyme-linked immunosorbent assay (ELISA). Malondialdehyde (MDA) was estimated by the thiobarbituric acid assay. Estimation of reduced GSH level was done using 5, 5’-dithiobis nitro benzoic acid assay. The comet assay was performed according to the method of Singh et al. (13) to evaluate the in vivo genotoxic potential of TiO_2_ nanoparticles in rats using the single-cell gel electrophoresis. Cells fixed in agarose were lysed to form nucleoids containing the DNA material. Electrophoresis at high pH results in comets which were detected by fluorescence microscopy. Based on the integrity of the comet tail and the head, we determined the number of DNA breaks. 

### Statistical analysis

Data were analyzed using Statistical Package for the Social Sciences software (SPSS version 22.0, IBM, USA). Differences between multiple means (quantitative variables) were evaluated by one way ANOVA test, followed by LSD. A P<0.05 was considered statistically significant.

## Results

**Fig.1:**
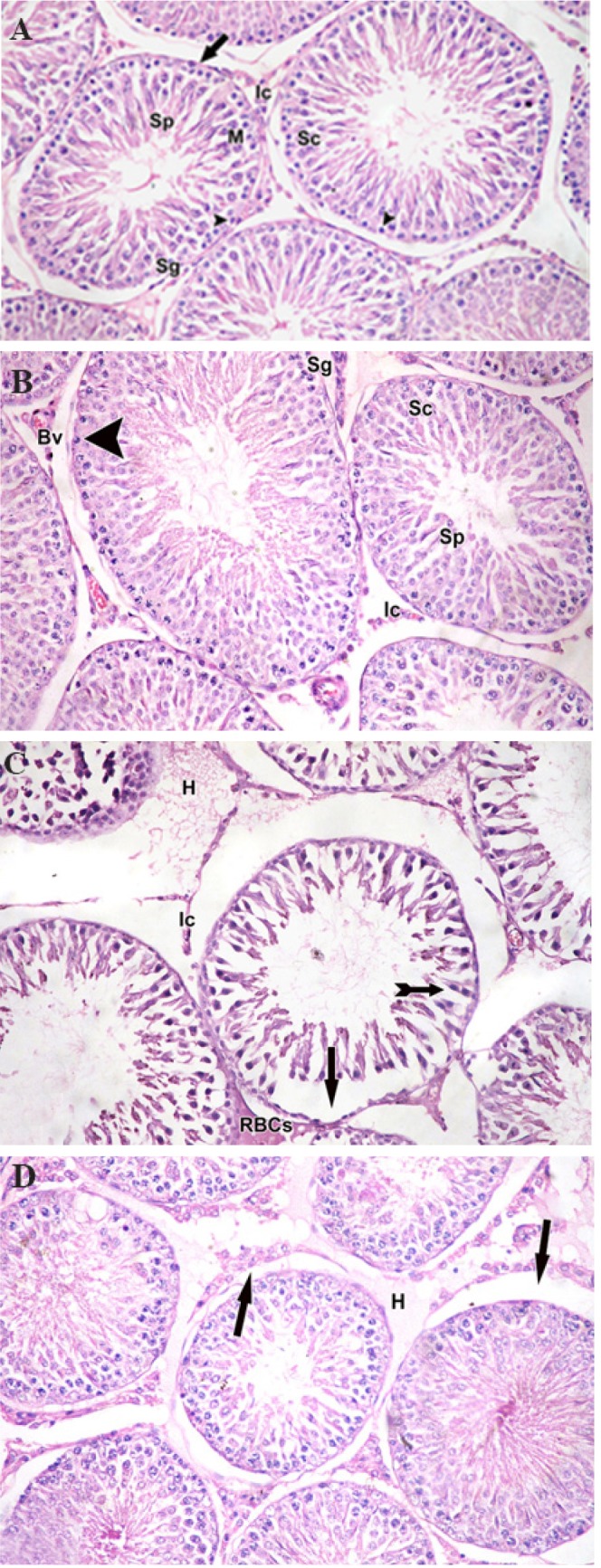
Photomicrograph showing histology of seminiferous tubules. A. Group I, II, and B. Group III showing normal seminiferous tubules lined by spermatogonia (Sg) close to the basal membrane (arrow), spermatogenic cells (Sc) with many mitotic figures (M) and Sertoli cells (arrow head). Seminiferous tubules lumen containing spermatid (Sp) with normal interstitial tissue (Ic) in between, C. Group IV showing marked disorganization, spermatogenic cells with dark pyknotic nuclei (tailed arrow), interstitial cells (Ic), basement membrane separation in many areas (arrow), extensive area between seminiferous tubules, hyaline exudate (H), and extravasation of blood (RBCs) in the interstitium, and D. Group V showing: separation of basement membrane of seminiferous tubules (arrow) and hyaline exudate (H) in the interstitium (H&E: ×200).

Macroscopic examinations of the left testis in terms of color, testis to body weight ratio, and infarction of treated groups revealed no significant changes compared to the control group. Histopathological examination of groups I, II, and III showed the same histological features without any abnormal histopathological finding such as dark nuclei, hyaline fluids, and blood extravasation into the interstitial spaces (Fig .1A, B). Meanwhile, histological examination of group IV showed disorganized seminiferous tubules, spermatogenic cells with dark pyknotic nuclei, separation of basement membranes, hyaline fluids, vacuolation, and extravasation of blood in the interstitial tissue. Moreover, some tubules showed thin layers of spermatogonia and sperms (Fig .1C). In Group V, there was a minimal separation of basement membranes with hyaline exudates in the interstitium (Fig .1D). 

**Fig.2:**
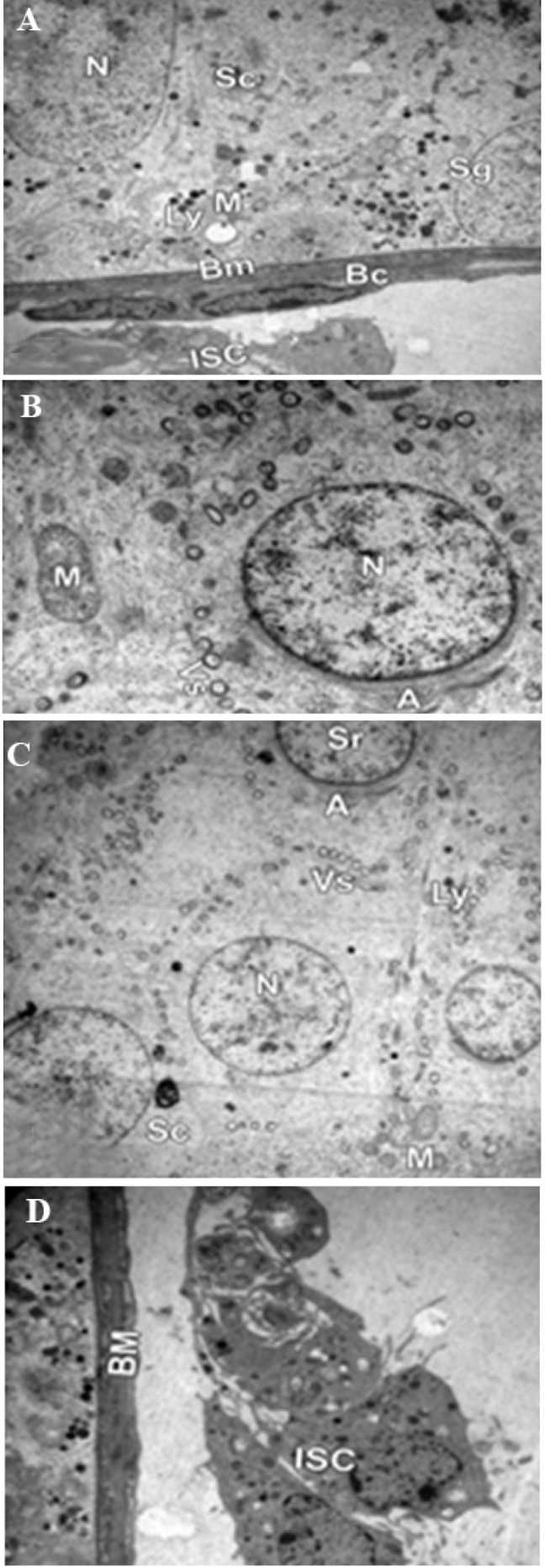
Photomicrograph showing electron microscopy of a seminiferous tubule. A, B. Group I, II ×4000, ×8000, C, and D. group III ×4000, showing spermatogenic cells (Sc) with its euchromatic nucleus (N). The cytoplasm contains mitochondria (M) and lysosomes (Ly); spermatogonia (Sg) resting on the basement membrane (Bm) with adjacent blood capillary (Bc) and interstitial cell (ISC); and a spermatid (Sr) with its acrosomal cap (A) and numerous vesicles (Vs).

**Fig.3:**
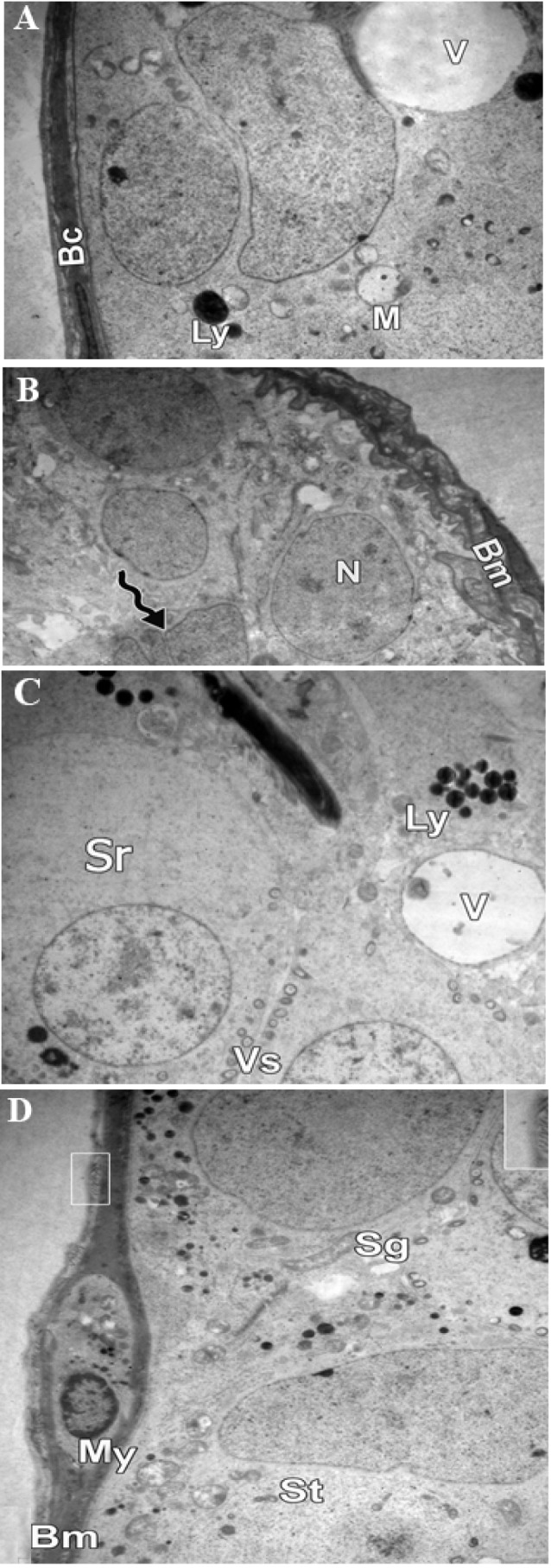
Photomicrograph showing electron microscopy of a seminiferous tubule. A, B. Group IV ×4000 showing irregular and thickened basement membrane (Bm), multi-folded euchromatic nuclei (N) with marked indentation (waved arrow), marked cytoplasmic vacuolation, mitochondria (M), lysosomes (Ly) and blood capillary (Bc), C, and D. Group V ×4000 showing vacuolation (V), spermatogonia (Sg) early spermatids (Sr) with their euchromatic nuclei and numerous vesicles (Vs), flattened myoid cell (My) and collagen fibers deposition (square) in the basement membrane (Bm).

**Fig.4:**
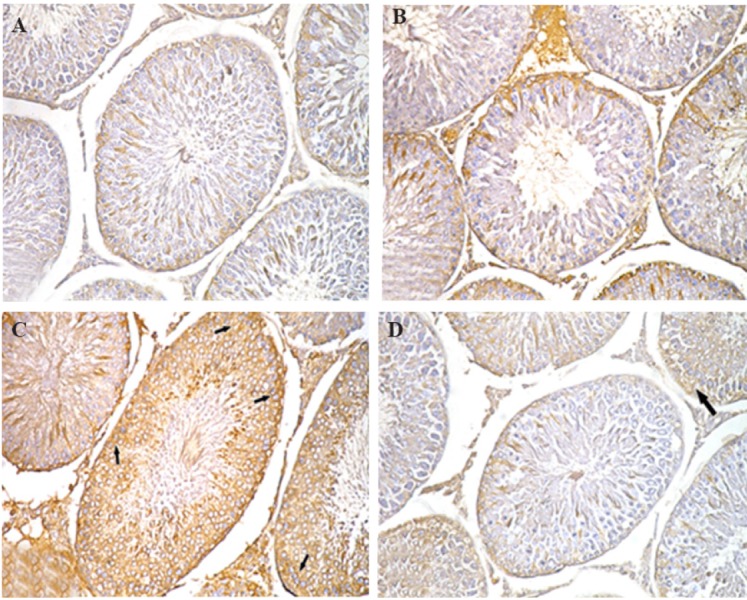
Photomicrograph showing immunohistochemistry of seminiferous tubules. A. Group I, II, B. group III showing weak TNF-a immuno-reactivity, C. Group IV showing strong positive TNF-a immunoreaction (arrow), and D. Group V showing decreased TNF-a immuno-reactivity (×200).

Using TEM, groups I, II, and II showed normal ultrastructures including normal seminiferous tubules lined with spermatogonia close to the basal membrane, spermatogenic cells with many mitotic figures and sertoli cells (Fig .2A-D). Group IV revealed signs of inflammatory damage in the form of thickened irregular wavy basement membrane with collagen fiber deposition, many abnormal multi-folded euchromatic nuclei with marked indentation, marked cytoplasmic vacuolation, and swollen mitochondria (Fig .3A, B). Cytoplasmic vacuolations with mild deposition of collagen fibers in the basement membrane were observed in spermatid and spermatogonia cells in group V (Fig .3C, D). Groups I, II, and III showed a relatively low TNF-a immunoreactivity (Fig .4A, B). On the other hand, a strong positive TNF-a immunoreaction was detected in group IV (Fig .4C) compared to group V which showed weaker immunoreaction (Fig .4D). 

There was an insignificant difference in total testosterone level among groups I, II, and III. However, the total testosterone level of group IV displayed a significant decrease compared to groups I, II, and III. Total testosterone level was significantly decreased in group V compared to groups I, II, and III . Meanwhile, there was a significant increase in total testosterone level of group V compared to group IV (Table 1). GSH values were insignificantly different among groups I, II, and III. However, there was a significant decrease in GSH level of group IV when compared with groups I, II, and III. Also, there was a significant increase in GSH level of group V compared to groups I, II, and III. Furthermore, there was a significant increase in GSH level of group V compared to group IV (Table 1). Additionally, there was a significant increase in MDA level of group IV compared to groups I, II, and III in addition to a significant increase in MDA level of group V compared to groups I, II, and III. On the other hand, there was a significant decrease (P<0.05) in MDA level of group V compared to group IV (Table 1).

**Table 1:** Total testosterone, serum reduced glutathione (GSH), and serum malondialdehyde (MDA)


Biochemical parameters	Group I	Group II	group III	Group IV	Group V

Total testosterone (nmol/L)	23.8 ± 8.8^bc^	21.2 ± 8.6^bc^	26.2 ± 8.8^bc^	0.38 ± 0.0^ac^	15.4 ± 6.0^ab^
GSH (nmol/L)	50.2 ± 4.0^bc^	49.1 ± 3.9^bc^	52.8 ± 4.7^bc^	31.8 ± 5.7^ac^	61.9 ± 3.5^ab^
MDA (nmol/L)	74.9 ± 3.5^bc^	75.0 ± 3.9^bc^	73.9 ± 3.4^bc^	136.3 ± 21^ac^	85.7 ± 6.6^ab^


Values are referred as mean ± SD. P<0.05 was considered statistically significant. ^a^; significance with group I ,II and III, ^b^; significance with group IV, and ^c^; Significance with the group V.

**Fig.5:**
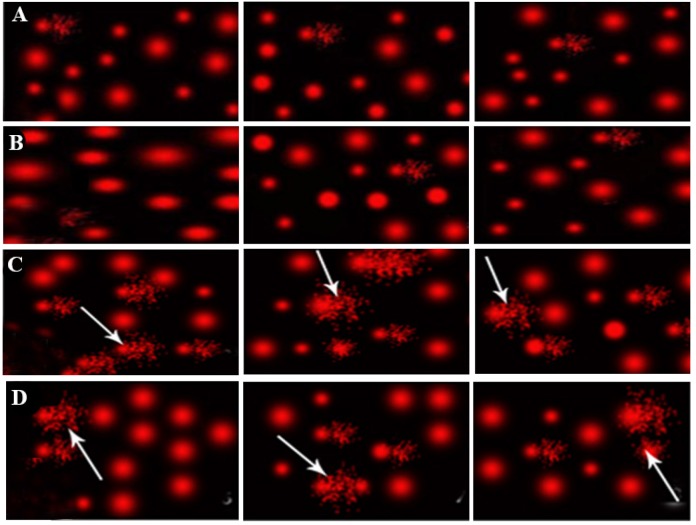
Photomicrograph of comet test showing nuclei of testes cells. A. Group I, II, B. group III showing almost normal condensed type nuclei and undamaged cells, C. Group IV showing abnormal tailed nuclei and damaged cells (white arrow), and D. Group V showing less number of abnormal tailed nuclei and damaged cells.

According to comet assay results, cells nuclei of group IV showed a significant increase in percentage (%) of tailed nuclei, tail length, tail DNA% and unit tail moment compared to groups I, II, and III. Co-administration of NAC and TiO_2_ nanoparticles caused a significant decrease in DNA damage parameters in group V compared to group IV. However, there was a significant increase (P<0.05) in % of tailed nuclei, tail length, tail DNA % and unit tail moment of group V compared to groups I, II, and III (Fig .5).

## Discussion

Prevalent applications of nanomaterial cause a huge potential for human exposure to these compounds. However, many experts and establishments have upstretched the environmental and toxicological concerns regarding nanotechnology (14). TiO_2_ nanoparticles have the ability to drift through diverse paths and accumulate in body tissues, which may lead to inflammation and apoptosis, resulting in various organ damages. The present study showed that TiO_2_ nanoparticles induced several histopathological alterations in the testes compared to the control group. Administration of NAC along with TiO_2_ nanoparticles showed improvements with minimal alterations in seminiferous tubules. Gao et al. (7) showed that nanoparticles-induced testicular injury and inhibition of spermatogenesis may attribute to changes in male sex hormone levels and testicular gene expression.

Our results are supported by data reported by Takeda et al. (8) which showed that exposure to TiO_2_ nanoparticles resulted in disrupted seminiferous tubules and tubule lumens with few mature sperms. Moreover, They observed aggregates of nanoparticles in Leydig’s cells, sertoli cells, and spermatids. Guo et al. (15) demonstrated a reduction in sperm density and motility in mice injected with TiO_2_ nanoparticles. On the other hand, Wang et al. (16) reported no abnormal pathological changes in the testes following acute toxicity induced by nano-sized TiO_2_ particles. The inconsistencies among these studies may be due to the differences in experimental conditions such as the animal model as well as administered dose, exposure duration, and the physicochemical characteristics of TiO_2_.

In the present study, examination of the testis sections of TiO_2_ nanoparticles-treated group revealed signs of inflammatory damage in the testicular tissue. For instance, administration of NAC along with TiO_2_ nanoparticles showed partial improvement in testicular tissue, which was found by histological and immunohistochemical examination. However, cytoplasmic vacuolation was still observed with mild collagen fibers deposition in the basement membrane. From an ultrastructural point of view, variable sized intercellular spaces were observed. El Ghazzawy et al. (17) stated that intercellular spaces represented advanced degenerative alterations damaging the cell membrane integrity as a result of oxidative stress. ROS induce oxidative phosphorylation of cell membranes resulting in interruption of the integrity of the intercellular junctional complex. These results are in accordance with those reported by Fouad et al. (18) which were obtained based on electron microscopy investigation of testicular tissues exposed to ROS and inflammatory cytokines measurements.

Co-administration of NAC and TiO_2_ nanoparticles led to a reduction in TNF-a immunoreactivity. Our results are in accordance with those indicated by Park et al. (19). Furthermore, there was a significant increase in testosterone level of the NAC+TiO_2_-treated group. A previous study found that sex hormone balance in the male reproductive system was disrupted by TiO_2_ nanoparticles exposure as the amount of testosterone was greatly reduced which led to suppressed spermatogenesis (7). Furthermore, EL-Sharkawy et al. (20) detected lowered testosterone levels in rats administered with TiO_2_; authors stated that reduction in testosterone secretion may be due to the high level of NO, which led to hypospermatogenesis, testicular inflammation, and disturbance of gonadotropin-releasing hormone secretion.

Co-administration of NAC and TiO_2_ resulted in a significant increase in GSH. These results showed a time-dependent reduction in GSH level in TiO_2_ nanoparticles-treated rats. Similar findings were reported by Long et al. (21) who observed GSH exhaustion and an upsurge in the lipid peroxidation levels after exposure to TiO_2_ nanoparticles. ROS generation was suggested as a probable mechanism involved in the toxicity of nanoparticles (5). Jeon et al. (22) speculated that a part of the ROS generation may be due to the catalytic properties of nanosized-TiO_2_. GSH level was remarkably decreased in the TiO_2_-treated group. However, it is worthy to say that GSH level was higher in NAC and TiO_2_ treated-group compared to NAC-treated group which was supposed to be decreased by TiO_2_. This may be explained by the ability of NAC to induce antioxidant effects in injury models rather than normal models (11, 23).

There was a significant rise in MDA level in TiO_2_ nanoparticles- treated group compared to the group treated with NAC+TiO_2_. However, MDA levels in NAC+TiO_2_ nanoparticles-treated group were significantly higher than those of the control group. Significant changes in MDA levels suggest that induction of pathological lesions is probably mediated through the oxidative stress enhanced by the dumped nanoparticles. These results were consistent with those reported by Attia et al. that showed a time- dependent significant release of oxidative stress in the liver as evident by increased MDA and reduced GSH levels (24). Furthermore, Gurr et al. (5) revealed an exponential increase in the MDA production caused by TiO_2_, and they attributed this increase in lipid peroxidation to excessive ROS generation.

The comet assay is a broadly used assay for investigation of DNA damage and repair, genotoxic properties of chemicals and pharmaceuticals, environmental biomonitoring, and also human monitoring. However, comet assay has been used for determination of the toxicity of highly reactive nanoparticles and several studies used it to test the potential toxicity of manufactured nanoparticles by assessing DNA strand breaks or oxidative DNA lesions (25). In the present study, results of *in vivo* comet assay showed that oral administration of TiO_2_ resulted in an increase in DNA damage in the testes. These results are in accordance with those noted by Shukla et al. (26) indicating that TiO_2_ nanoparticles generate ROS and cause DNA damage and genotoxicity in mammalian cells. The direct association between ROS generation and oxidative DNA damage further proposes that oxidative stress can act as a significant path through which, TiO_2_ nanoparticles cause DNA damage. Previous studies showed that TiO_2_ nanoparticles caused DNA injury indirectly through inflammation (27) and generation of ROS (5).

Furthermore, TiO_2_ nanoparticles in aqueous suspension release free radicals which can result in DNA damage by oxidation, nitration, methylation or deamination reactions (28). Since TiO_2_ nanoparticles prompt inflammatory reactions and DNA injury, it was suggested that TiO_2_ nanoparticles act an indirect genotoxicity inducer as suggested by Dankovic et al. (29). Previous studies reported DNA damage caused by TiO_2_ nanoparticles using *in vitro* (5, 16) and *in vivo* comet assays (30). On the other hand, negative results were reported concerning TiO_2_ nanoparticles-induced DNA damage in studies using *in vitro* experiments (31) and *in vivo* comet assays (32). Tao and Kobzik (33) suggested that discrepancies among studies may be due to irregular TiO_2_ release, particle size, the extent of accumulation, and incubation circumstances, suggesting that additional studies should be done to determine the situations in which TiO_2_ nanoparticles genotoxicity arises.

NAC acts as an antioxidant through expanding the synthesis of endogenous GSH which is frequently exhausted as a result of augmented oxidative stress (23). Additionally, NAC performs as a direct scavenger of free radicals (34). Together, these antioxidant activities of NAC can attribute to guard against oxidative stresses. These results are consistent with those mentioned in El-Kirdasy et al. (35) study. The protective effects of NAC on testicular damage and dysfunction, were also demonstrated by other studies (36). NAC has been shown to have significant effects on testicular dysfunction. Consistent with the decrease in TNF-a immunoreactivity in the current study, Dick et al. (37) reported that NAC pretreatment stops TNF-a production in alveolar macrophages treated with nickel particles. Attia et al. (24) stated that co-treatment with NAC and TiO_2_ restored MDA and liver cells GSH levels. Furthermore, Xue et al. (10) detailed that NAC powerfully repressed ROS production in TiO_2_-treated cells and blocked nano-TiO_2_ induced lipid peroxidation, and apoptosis. The diminished level of DNA damage in nuclei of the testes following treatment with NAC was in accordance with results reported by Shi et al. (38) which showed that NAC administration suppressed the level of TiO_2_ nanoparticles-induced DNA injury in human lymphocytes. The suppressive effect of NAC on ROS formation in cells exposed to TiO_2_ was also noted by Xue et al. (10). Moreover, NAC showed significant effects on the volume and motility of semen by increasing the antioxidant level and reducing peroxide and oxidative stress index when compared to the control group, in a clinical trial. This was explained by NAC ability to diminish ROS and reduce the viscosity of the semen (39).

## Conclusion

Oral administration of TiO_2_ nanoparticles induced toxic effects and DNA damage in the testes and these adverse effects may be attributed to induction of oxidative stress. Administration of NAC along with TiO_2_ nanoparticles, protected against TiO_2_ damaging effect.
